# Understanding Experiences of Diabetes Distress: A Systematic Review and Thematic Synthesis

**DOI:** 10.1155/2024/3946553

**Published:** 2024-11-14

**Authors:** Louise Anne Morales-Brown, Guillermo Perez Algorta, Yakubu Salifu

**Affiliations:** Department of Health Research, Health Innovation One, Sir John Fisher Drive, Lancaster University, Lancaster LA1 4AT, UK

**Keywords:** diabetes mellitus, psychological distress, systematic review

## Abstract

**Background:** Diabetes distress is a common emotional issue for those living with diabetes, which has the potential to negatively impact well-being, management behaviors, and HbA1c levels. These implications have led to diabetes distress becoming an important consideration in diabetes healthcare and management. Nonetheless, discussions remain ongoing on how to best conceptualize this experience. Recent research has attempted to enhance conceptualization by considering the underlying emotional mechanisms that may underpin the highly contextualized experience of diabetes distress. Qualitative insights can further add to these understandings; however, the research in this remit is yet to be systematically reviewed. This review therefore sought to add to the growing body of literature attempting to better conceptualize diabetes distress and the underlying mechanisms that may contribute to this experience. A secondary aim was to leverage this understanding to consider ways to improve patient–healthcare interactions.

**Methods:** A qualitative systematic review and thematic synthesis was undertaken. Eligible studies were identified through PsycINFO, MEDLINE, CINAHL, and EMBASE databases from November 2020 to May 2021. Study quality was assessed using the McMaster Critical Review Form.

**Results:** Nineteen papers were included in the review. The analysis resulted in seven descriptive themes which contributed to three analytical themes: (1) threatened autonomy, (2) sense of helplessness, and (3) negative sense of self. These results highlight that a major area underpinning experiences of diabetes distress is not feeling in control.

**Conclusions:** Consideration should be given to how psychological factors, such as locus of control and learned helplessness, may constitute underlying mechanisms impacting emotional regulation in those experiencing diabetes distress. Clinicians should consider including and leading discussions around distress during appointments, as well as using approaches that promote patient autonomy and empowerment.

## 1. Introduction

Diabetes distress, diabetes-related distress, and diabetes-specific distress are terms that describe feelings of frustration and worry that often accompany living with and managing diabetes mellitus [1]. Although a person experiencing diabetes distress may demonstrate depressive and anxious symptoms, diabetes distress is not considered a psychiatric illness but a stress response to living with the condition [2].

Elevated levels of diabetes distress have been associated with poorer self-management behaviors and increased hemoglobin A1c (HbA1c) in both adults with Type 1 diabetes (T1DM) and Type 2 diabetes (T2DM) [3–5]. Thereby, increasing the risk of developing health complications associated with extended periods of hyperglycemia. In further support of this relationship, a reduction in levels of diabetes distress showed modest improvements in self-management behaviors and a reduction in HbA1c [6]. Consequently, diabetes distress has accumulated attention due to its negative psychological implications and its potential for negative physical health outcomes.

Several self-report surveys have been developed to measure diabetes distress, namely, the Problem Areas in Diabetes (PAID) [7], the Type 1 Diabetes Distress Scale (T1-DDS) [8], and the Diabetes Distress Scale (DDS) [9]. The former two were standardized primarily in adults with T1DM, while the latter was standardized in those with T2DM. While all surveys yield an overall distress score, the T1-DDS and DDS yield measures in several subcategories that represent areas of diabetes distress identified in the respective cohort.

These scales successfully capture many facets of diabetes distress and are useful as a starting point in indicating possible areas of distress, the severity of distress, and facilitating patient–clinician discussions [10]. However, it has been argued that these existing measures lack content validity and do not provide a comprehensive assessment of this phenomenon [11]. This may in part be due to diabetes distress being a contextualized experience, varying by factors such as age, culture, type of diabetes, and medication type [12]. For example, adults with T2DM who manage their condition with insulin instead of tablets experience diabetes distress related to hypoglycemia and powerlessness [13]. Nonetheless, these sources are exclusively mentioned in the T1-DDS and not in the DDS.

Variations in sources of diabetes distress, coupled with the lack of content validity in current measurement tools, highlight the difficulties in conceptualizing diabetes distress. To overcome this, attempts have been made to define it as a distinct concept [11, 14, 15]. As part of this, research efforts have sought to understand what may underpin the experience of diabetes distress, focusing largely on emotional mechanisms [16–18]. For example, Fisher et al. identified that increased diabetes distress is associated with three facets of poor emotional regulation: self-judgement of emotions, impulsive reaction to emotion, and a lack of awareness of emotional experiences related to diabetes [18].

In order to contribute to the growing literature seeking to better conceptualize diabetes distress and the underpinnings of this phenomenon, it warrants consideration of the qualitative literature surrounding patient experiences. Qualitative literature can provide detailed insights that facilitate the conceptualization of phenomena [19]. Analyzing rich descriptions of patient experiences may uncover underlying mechanisms that drive certain experiences, in this instance, diabetes distress. Not only could this lead to considerations for current and future measurements of diabetes distress but approaches to interventions focused on reducing diabetes distress as well.

Understanding patient experiences of diabetes distress may also benefit patient–clinician interactions. One of the primary mediums for addressing diabetes distress in the first instance is a person-centered conversation [20, 21]. Despite this, healthcare professionals feel ill-equipped to address issues of well-being in diabetic patients [22, 23]. Research exploring patient experiences can often help clinicians gain better insight into patient perspectives and facilitate an empathetic understanding [24]. Furthermore, information regarding patient experiences can help nurses make sense of certain behaviours [25]. This is pertinent where diabetes distress may reveal itself as poor self-management behaviors and higher glucose levels.

To the researcher's knowledge, no systematic review exists exploring patient experiences of diabetes distress. Accordingly, this systematic literature review seeks to answer the following question: *How is diabetes distress experienced in people living with diabetes mellitus?* The ensuing aims are threefold: (1) to consider what mechanisms may underpin the experience of diabetes distress, (2) to contribute to the conceptualization of diabetes distress, and (3) to provide evidence that may better inform patient–clinician interactions. To achieve this, the available qualitative literature on patient experiences of diabetes distress will be aggregated and analyzed in line with the processes of thematic synthesis.

## 2. Methods

The thematic synthesis was registered within the International Prospective Register of Systematic Reviews (PROSPERO) database from the University of York (registration number: CRD42020218468). Results have been presented in accordance with the reporting guidance set out in the enhancing transparency in reporting the synthesis of qualitative research (ENTREQ) statement.

### 2.1. Theoretical Framework

This thematic synthesis is underpinned by a critical realist approach. Critical realism infers a realist ontology, implying that a reality exists independent of human conceptualization [26]. Knowledge of this reality is stratified into three layers: (1) a real domain where generative mechanisms enable or constrain actions and experiences, (2) an actual domain that consists of events and their effects that have been caused by the real domain, and (3) the empirical domain which refers to observable actions and experiences [27].

A thematic synthesis is a form of metasynthesis that enables the production of new concepts in a transparent way while staying closely aligned with the findings of primary studies [28]. Thematic synthesis aligns with critical realism as both seek to comprehend how lived experiences may interplay with objective reality [29, 30]. It is therefore well suited to consider an array of patient experiences of diabetes distress and the mechanisms that may contribute to this experience.

### 2.2. Search Strategy

Search terms were defined using the SPIDER framework (Table 1), a tool designed to identify relevant qualitative and mixed-method studies [31]. The search strategy of quantitative systematic reviews exploring diabetes distress was also examined to ensure appropriate synonyms and terms were incorporated. The author also consulted with librarians to refine the search strategy.

From 22 November 2020 to 31 May 2021, the author independently searched PsycINFO, MEDLINE, CINAHL, and EMBASE databases. These databases were selected as they catalogue literature from biomedical and psychological sciences.  Table 2 demonstrates the full search conducted on CINAHL, alongside database-specific terms.

### 2.3. Selection Criteria

The inclusion criteria for this review were studies that (i) explored experiences of diabetes distress or those that incorporated substantial data from questions or themes exploring the adverse emotional aspects of living with diabetes, (ii) stemmed from the perspective of participants living with T1DM or T2DM, (iii) used qualitative methods of data collection and included sufficient reporting of the qualitative data, either as standalone studies or as a distinct part of mixed-methods studies, and (iv) were in English.

Excluded studies were (i) those where the aim of the research was to explore comorbid physical or mental health problems alongside diabetes (e.g., diabetes and depression or diabetes and cancer), (ii) participants with prediabetes or gestational diabetes, (iii) articles that limited the experience to a specific context (e.g., diabetes management in school), (iv) responses to treatments attempting to reduce diabetes distress, (v) commentaries and discussion pieces lacking original data, (vi) systematic and other literature-based reviews, and (vii) abstract only or conference pieces.

### 2.4. Quality Appraisal

Papers were assessed using the McMaster Critical Review Form for Qualitative Studies [32]. This tool caters to a range of research designs such as phenomenology, ethnography, and grounded theory. Moreover, as well as considering the researcher's methods and analytical approaches, it also considers research integrity by assessing concepts of trustworthiness [33]. Despite appraising all papers, studies deemed of lower quality were included in this review, as even low-quality publications have the potential to generate new insights based on participant accounts [34].

### 2.5. Data Extraction and Synthesis

Papers were uploaded onto NVivo 12 software and read multiple times to gain familiarity. The papers were then analyzed in line with Thomas and Harden's approach to thematic synthesis [28]. This involved (1) the author conducting line-by-line inductive coding of each paper's result section. Where appropriate, interpretations of data identified elsewhere (e.g., Appendix or Discussion sections) were also coded. At least one code was given to all statements relating to diabetes distress or adverse emotional experiences associated with living with diabetes. (2) Descriptive themes were developed by examining similarities and differences between the codes and aggregating them under appropriate headings. This involved grouping the initial codes across papers to provide an overarching code. Similar overarching codes were then combined to create descriptive themes (see Supporting Information 1, for example). (3) The author created analytical themes from the descriptive themes, going beyond the content of the original studies to address the review question. The generation of the analytical themes involved the researcher identifying connections between descriptive themes that made sense of people's experiences of diabetes distress. The latter steps move from a data-driven approach to one that is theory-driven [28]. This draws parallels to critical realist principles of moving from observations to theorising mechanisms that explain a particular phenomenon, in this instance diabetes distress [35].

### 2.6. Reflexivity

As a person living with T1DM, the primary author harbors their own experience and understanding of diabetes distress. Throughout the review, a reflexive journal was maintained to assist the primary author in recognizing their biases and to ensure transparency by acting as an audit trail [36]. To further minimize biases, the author regularly engaged in conversations with their coauthors and shared initial drafts of emerging themes.

## 3. Results

**Figure 1:**
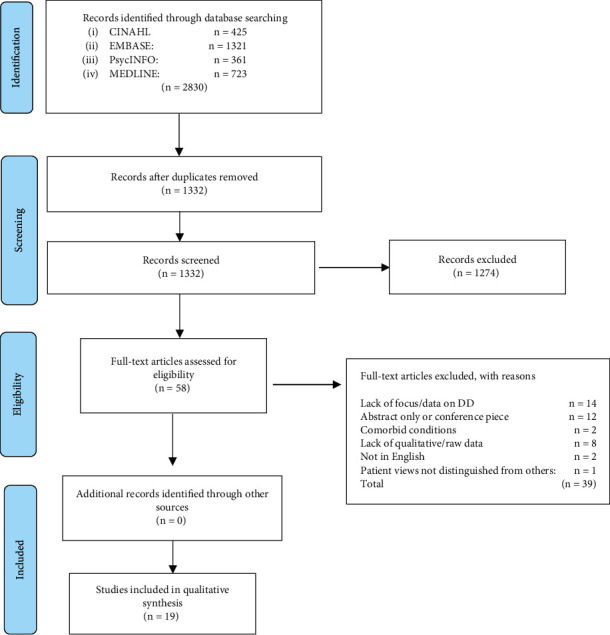
PRISMA flow.

The search retrieved 2830 papers; 1498 were duplicates. The author first reviewed titles and abstracts against the inclusion and exclusion criteria, eliminating 1274 papers, and then a further 39 through full-text review (see Figure 1). No further papers were identified through citation tracking or examining the reference lists of included papers. The final number of papers included in this review is *n* = 19.

### 3.1. Characteristics of Included Studies

One large-scale study included in this review involved 8596 participants, 7228 with T2DM and 1368 with T1DM. The average age of participants in this study was 57.0 years, and the mean duration of diabetes was 9.0 years. The remaining papers included a total of 634 participant experiences (404 female, 223 male, and 7 unknown) from across the world, but largely from Western countries. The type of diabetes (reported in *n* = 17 studies) was predominantly T2DM (331), followed by T1DM (297). The mean age range (reported in *n* = 13 studies) was 12.28–63.3 years, and age ranges in studies not reporting means or medians (reported in *n* = 6 studies) were between 21 and 87 years. The mean duration range of the onset of diabetes (when reported, *n* = 13) was between 5.6 and 19.75 years.  Appendix 1 provides a summary of the 19 papers included within this thematic synthesis. Two papers draw on the same data set [37, 38].

The results from the quality assurance are outlined in Table 3. Few studies reported on their theoretical perspectives or preconceived biases held by the researcher. Additionally, few studies noted as to whether sampling was done until redundancy in the data was met. Three studies met all four of the components for overall rigor.

### 3.2. Analytical Themes

The analysis resulted in seven descriptive themes (Table 4), which largely provide a high-level overview of the sources of diabetes distress as perceived by participants.

**Figure 2:**
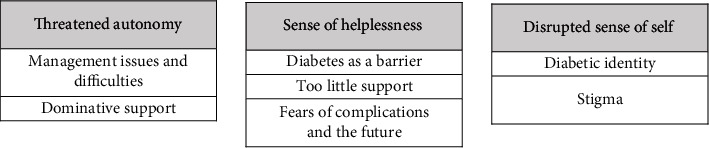
Analytical themes and underpinning descriptive themes.

The descriptive themes contributed to three analytical themes to develop an understanding of what underpins the experience of diabetes distress: (1) threatened autonomy, (2) sense of helplessness, and (3) negative sense of self.  Figure 2 shows how the descriptive themes sit beneath the analytical themes.

#### 3.2.1. Threatened Autonomy

This analytical theme focuses on how management difficulties and dominative support seemed to negatively impact a person's sense of autonomy. All studies in this review, to some degree, uncovered experiences and sources of diabetes distress that could feed into this theme.

##### 3.2.1.1. Management Issues and Difficulties

Control plays an integral part in diabetes management where the goal of treatment is to keep glucose levels as near to a healthy range as possible. Participants often found the process of controlling their glucose levels relentless: “It is a mental burden to take care of the disease all the time” (T1DM, p26) [39]. However, the complex and unpredictable nature of the condition often made control difficult to achieve, leading to incidents of hypo- and hyperglycemia: “My sugar will go anywhere from 109 to 400 in a matter of 10 minutes. Last night, I went to sleep and it was 396. This morning, it was 62…It's hard because I don't know exactly what makes my sugar go as crazy as it does” (T2DM, p6) [13]. As hypo- and hyperglycemic episodes can have life-threatening consequences, participants feared making a mistake that could lead to such an event: “I panic a lot because one night my blood sugar dropped in the middle of the night and I ended up like having a seizure” (T1DM, p550) [40].

The stress of achieving in-range glucose levels was also apparent in receiving HbA1c results, an index often used by clinicians to assess a person's level of control over their condition: “And I was like really nervous to go in [to the clinician's office] and like have to like say in front of my mom maybe like ‘oh my God, your son's A1c is so high.' (T1DM, p549) [40]. When not in the target ranges, participants often saw this as a failure on their behalf and felt disappointed or guilty: “If you take that reading and you find that you're 400 and above or 300, it's like denial and then it hits you in the face” (T2DM, p7) [13].

The responsibility of controlling a condition which at times could be erratic caused a sense of futility among participants: “No one understood how frustrating it can be sometimes when you're doing your damndest to get it right and it's not working” (T1DM, p6) [41]. As such, instead of feeling as if they were controlling the condition, this led some participants to feel as if the condition was controlling them: “I wake up in the morning and stick myself and this little meter dictates how I am going to run the rest of my day” (T2DM, p152) [42].

##### 3.2.1.2. Dominative Support

The second instance where autonomy felt threatened was in interactions with clinicians and family. Comments from healthcare professionals could sometimes be interpreted as authoritative: “Doctors' advice to women tended to be in the form of injunctions — ‘be careful', ‘take care', ‘go on a diet and lose weight', ‘watch what you eat' (T2DM, p294) [37] and when participants attempted to input into their regiment, they felt ignored or not taken seriously “I feel like he's brushing me off, that sort of feeling. Because I want to know if I could reduce the dosage for this medication, but it feels like he doesn't take my question seriously” (T2DM, p966) [43]. By discouraging patient input into their condition, these interactions invalidated patient autonomy and diminished empowerment.

Familial interactions could also be perceived as controlling and overbearing. “That's why I don't want to share a lot because then they start getting in the action and doing things. They think that it's their duty to do something to help. And that frustrates me” (T2DM, p296) [37]. Participants found these interactions unwelcome and intrusive, leading to familial tensions and feelings of guilt and bitterness. In some instances, it also led to defiance in an attempt to regain control: “Like she's trying to command me to do stuff but yet being a dumb sailor, I won't do it. I don't like being told what to do” (T2DM, p89) [44].

#### 3.2.2. Sense of Helplessness

In all studies, participants discussed feeling limited, receiving inadequate support, and fears of complications as sources of diabetes distress. These were encapsulated under the analytical theme of a sense of helplessness, whereby participants felt trapped and powerless as a result of living with the condition.

##### 3.2.2.1. Diabetes as a Barrier

Participants expressed sentiments of helplessness through the limitations diabetes imposes on their lives: “There's limits to everything” (T2DM, p294) [37]. For some, the limitations that diabetes imposed created a sense of disadvantage: “Stuff like college work can get affected and going out with your friends. You're concentrating too much on this element and the rest of yourself is neglected a bit” (T1DM, p6) [41]. This sentiment was echoed when participants discussed how the side effects of diabetes, such as fatigue, could also act as a hindrance to day-to-day activities: “everything has been reduced … I don't have enough energy anymore” (T2DM, p295) [37].

For others, there was a sense of deprivation from missing out on activities that would otherwise be considered normal: “There was this party, and like, they were having this cake that like, they said it had a lot of sugar in it and I don't want to, like, raise it up a lot, and I didn't go” (T1DM, p548) [40]. Reasons as to why participants felt limited by their condition were often personal, with some rooted in contextual factors such as culture or religion: “She said it's for Allah's sake you're fasting. When she sees others fasting and she can't do it herself she feels bad” (T2DM, p308) [45].

##### 3.2.2.2. Too Little Support

Feelings of helplessness were often a by-product of interactions with participant's clinicians or healthcare professionals. Appointments were often rushed and felt impersonal, “You get pushed in; you get pushed out. That's all” (T2DM, p6) [13] and inconsistent advice could lead to confusion about the regiment, “Every doctor has his/her opinion when I ask about sugar. One doctor told me that it is okay to drink a little sugar, while some strongly prohibit it” (T2DM, p117) [46]. There was also a belief that healthcare professionals gave too much attention to biomedical aspects of care, “They just have a quick look at the file and look at numbers without discussing any of the issues with you” (T1DM, p9) [41], despite participants wanting to receive emotional support during these interactions, “It would be best to have someone who understands [health professional]. I'll vent to them, then console me, then my heart will be calmed down. There's no such people!?” (T2DM, p967) [43].

A similar sense of invalidation was seen in instances when participants felt unsupported by their family: “My sister's kid has got Type 1, and it's like ‘Aw you're not as bad off as my children'. Like what a cruel thing to say. They maybe think I'm putting it on a bit, I mean they have no idea….so now I just help myself and never let on. There's no support there, no, not at all” (T2DM, p1674) [47]. The lack of empathy and understanding from family contributed to a sense of loneliness, “when you've got something, it's only you that has it” (T2DM, p25) [38] and likewise to clinical interactions, some participants felt an absence of consideration for the emotional side of living with diabetes, “I think my family do not really understand the mental side (T1DM, P9) [41].

##### 3.2.2.3. Fears of Complications and the Future

Helplessness also underpinned the concerns that participants held related to the development of complications from poorly controlled diabetes: “That's the fear, the complications of diabetes are awful” (T1DM, p7) [41]. Some felt this apprehension regularly: “When I eat something right away, I worry [that] I'm eating the wrong thing, my sugar's going to be up, I'm hurting myself. What is it doing? Is it causing nerve damage right now? Is it in the future going to do something?” (T2DM, p9) [13]. The consequences of these complications were also a cause of concern, “It worries me a lot and I sometimes do not sleep; if I have something [a health complication] I cannot work” (T2DM, p860) [48]. For those who had already developed complications, such as amputations, these fears were confirmed and led to a sense of hopelessness about the future: “I would rather be dead, to be honest” (T1DM, p2470) [49].

Linked to complications, but highlighted as a distinct complaint, was the fear of early mortality: “Damn it! I am going to die soon; I am going to die young” (T2DM, p859) [48]. For children and adolescents, this fear was less pronounced; however, as the age of participants increased, this became a growing area of concern. “Interviewees felt that their concerns about the future increased as they transitioned through their twenties” (T1DM, p7) [41]. The fear of death was therefore more prominent in adulthood and those with family members who had died as a result of diabetic complications: “I started out very nervous about it because my father was a diabetic, and he died a terrible death. He died because he had strokes and other things that killed him at the age of 65” (T2DM, p94) [44].

#### 3.2.3. Disrupted Sense of Self

In 15 out of 19 papers, participants discussed how diabetes can negatively impact a person's identity [13, 37–41, 46–54] This could come from either an internal belief, such as the extent people felt defined by their diabetes, but also externally through societal views and discriminatory behavior. The other analytical themes of “threatened autonomy” and “sense of helplessness” also fed into this by acting as potential triggers which could exacerbate participant's negative perceptions.

##### 3.2.3.1. Diabetic Identity

Participants felt that diabetes was a large part of their identity. For those with T2DM, the condition was perceived as having negatively impacted their social roles and personality. This was often expressed in the form of reflections on life prediagnosis: “Its affected my confidence and I suppose I miss the way I used to be before. I just haven't got the confidence I used to have” (T2DM, p26) [38]. For these individuals, a comparison of who they were prior to their diagnosis was used as a benchmark for their identity and life postdiagnosis. Often, these changes were interpreted negatively.

For others, living with diabetes was synonymous with being sick, “they feared that peers would treat them like they were ‘sick'” (T1DM, p548) [40] or being seen as damaged, “You will always be diabetic, you are damaged in ways, your pancreas is damaged… it's getting your head around it all” (T2DM, p1672) [47]. Whether reflecting on their personality prediagnosis, how others define them, or how they define themselves, participants created a dichotomy between a life with and without diabetes. The latter was underpinned by health and positivity, while the former was interpreted as sickness and damage.

##### 3.2.3.2. Stigma

The negative beliefs participants held about themselves were reinforced by instances of stigma and discrimination. In some cases, participants experienced this directly, “one Metis woman was told ‘you are too fat, you eat too much'” (unspecified p329) [51]. However, participants also expressed distress related to broader societal interpretations of diabetes. Often, these interpretations perpetuated stereotypes associated with T2DM which blame the individual for developing the condition through consuming excess sugary foods or being overweight, “People are apt to make a fun of diabetes. For example, on TV, the disease is often used for laughs, such as “If you eat sweets so much, you will have diabetes!” (T1DM, p23) [39]. Such stigma also existed within the diabetic community, with people living with T1DM feeling the need to distinguish themselves from those with T2DM. “That's something that drives me crazy, Type 1 and 2 diabetes. It makes me so annoyed. Type 1 diabetes, you don't get it because you're overweight” (T1DM, p5) [41].

The awareness of the stigma tied to diabetes made participants secretive, hiding their diagnosis from others to avoid judgement: “I find it hard to express to people that I have diabetes, and I try to keep it hidden so that most people don't find out, because I feel like they will judge me” (T1DM, p547) [40]. In some instances, it also led to participants compromising self-management behaviors. “One woman explained that she did not take insulin in front of her boyfriend's parents because diabetes was viewed as a defect, which was unacceptable” (T1DM, p2470) [49]. The negative views and assumptions surrounding diabetes therefore contributed to self-stigma, causing participants to feel shame and self-consciousness.

## 4. Discussion

This thematic synthesis sought to explore how people living with diabetes mellitus experience diabetes distress so as to consider the mechanisms that may underpin this phenomenon and improve patient-healthcare interactions. Participants largely struggled with issues tied to feeling in control, and this was apparent in the themes “threatened autonomy” and “sense of helplessness.” Diabetes mellitus was interpreted as a dominating and pervasive condition, where attempts to control it were often seen as futile due to its fluctuant nature. Participants positioned themselves as powerless against the condition that consistently preoccupied their thoughts and lives.

Attacks on autonomy were also apparent in interactions with healthcare professionals and family members. Too little support was seen as inconsiderate, whereas too much was interpreted as intrusive or controlling. Participants found appointments to be impersonal and rushed, which is consistent with findings that have identified healthcare professionals as failing to adhere to a person-centered approach with diabetic patients [55]. As demonstrated in patient narratives, this instilled a sense of inadequacy and helplessness.

The final theme, “disrupted sense of self” emerged from participants viewing themselves through a negative lens, labelling themselves as “sick” or “damaged.” Lower levels of self-esteem have been associated with higher levels of diabetes distress [56], which is potentially reflected in the accounts of those included in this review. Participants' negative self-image were further exacerbated by instances of stigma, an issue that is prevalent among the diabetic community [57].

These findings are consistent with research suggesting that helplessness is also experienced by those with T2DM [13]. Although aspects of helplessness have been acknowledged in the T1-DDS under the subscale of “powerlessness,” this is omitted in the measurement for those with T2DM as it was not identified as part of their experience of diabetes distress [9].

These findings also lend themselves to wider discussions regarding the concept of autonomy in chronic illness, the definition of which has been critiqued for being too narrow and often synonymous with decision-making [58]. In chronic illness, where decisions around treatment and medicine may be limited, the focus of autonomy shifts to maintaining control over one's life despite the perceived restrictions of the condition [59].

### 4.1. Future Considerations

These insights have several practical implications that may warrant consideration. Despite wanting to discuss the emotional impact of living with diabetes [60], people living with diabetes feel disempowered during interactions with healthcare professionals. To overcome this, healthcare professionals could consider implementing techniques drawn from autonomy support. Within the remit of chronic disease management, this form of support involves acknowledging patients' perspectives, providing choices, responding to patients' self-care initiatives, and minimizing control of patients' self-care behavior [61]. This approach from family members has been shown to ameliorate the effects of diabetes distress in those with T2DM [62], and a similar effect has been observed with healthcare professional interactions with adults with T1DM [63].

Where issues around helplessness and loss of control seem tied to autonomy, those experiencing elevated levels of diabetes distress may demonstrate a strong external locus of control. Those with this perspective attribute outcomes to external factors such as circumstance, fate, or unfairness [64]. This belief may be associated with higher HbA1c levels [65, 66] a consequence identified in those presenting with high levels of diabetes distress [67]. Further research is warranted to understand how locus of control and elevated levels of distress may be associated.

When individuals repeatedly and over time experience a perceived absence of control over a situation's outcome, they may develop learned helplessness, a phenomenon where they believe they are unable to control or change the situation and, as a result, no longer attempt to try and change the situation [68]. Older research identified this behavior in diabetic youth with poor metabolic control [69], and the culminative negative experiences in diabetes management may lead to learned helplessness [70]. Similarly, to the locus of control, this may in part explain why higher levels of diabetes distress are associated with poorer self-management behaviors and an increase in HbA1c levels [3–5]. Future research should consider associations between levels of diabetes distress and learned helplessness, as this may be an area of importance to address in interventions.

These insights could compliment the research on diabetes distress and emotional regulation. External locus of control has previously been identified as an exacerbator of psychological distress [71–73] and a predictor of emotional dysregulation [74]. Similar findings have been highlighted in learned helplessness [75]. These concepts may therefore act as mechanisms underlying the difficulties in emotional regulation identified in those experiencing diabetes distress. Greater exploration is necessary to understand if and how these psychological factors influence this phenomenon.

### 4.2. Limitations

This synthesis carries several limitations. Firstly, while some studies in the search had an explicit aim to explore diabetes distress, others did not. Most notably, these tended to explore lived experiences of having diabetes or experiences of diabetes management. A pragmatic decision was made to examine the aims, interview questions, and analytical themes of these studies and only include those with themes related to emotional distress rooted in living with or managing diabetes. This risked not including every study that explored diabetes distress. Nonetheless, the goal of thematic synthesis is to be purposeful rather than exhaustive. As such, it is not necessary to locate every available study because the concepts drawn during the analysis will remain the same regardless of the number of studies [28].

Secondly, the inclusion criteria were restrictive, which may have led to missing relevant perspectives and variations in experiences. This decision was made to maintain a clear focus for the review. Future studies may therefore want to consider experiences of distress in cohorts such as those living with diabetes and comorbid physical or mental health conditions, prediabetes, or gestational diabetes [76].

Lastly, the majority of experiences included were that of females with T2DM, living in Western societies, and a disease duration of 5.6 years. Consequently, the interpretation of results may be skewed to represent the experience of this cohort. To further enhance understanding in this area, future research should consider experiences from non-western cultures and adults recently diagnosed with T1DM. Consideration could also be given to whether the underlying mechanisms identified in this review may also be associated with other metabolic disorders [77].

## 5. Conclusion

Despite these limitations, the present review contributes to a growing body of evidence attempting to both better conceptualize diabetes distress and improve patient–clinician interactions. Regarding the latter, clinicians should consider including and leading discussions around distress during appointments, as well as adopting approaches that promote patient autonomy and empowerment. In terms of the former, consideration should be given to how locus of control and learned helplessness may constitute underlying mechanisms impacting emotional regulation in those experiencing diabetes distress.

## Figures and Tables

**Table 1:** SPIDER search criteria.

S—Sample	People with Type 1 diabetes (T1DM) and Type 2 diabetes (T2DM)
Pi—Phenomena of interest	Diabetes distress, diabetes-related distress, and diabetes-specific distress.
D—Design	Qualitative approaches include but are not limited to interviews, focus groups, online forums, and visual explorations
E—Evaluation	Experiences of diabetes distress or adverse emotional reactions to living with diabetes
R—Research type	Qualitative and mixed methods

**Table 2:** Search strategy: CINAHL (Via EBSCO) search strategy, 8 November 2020.

**Search ID #**	**Query**	**Results**
S8	S7 AND S6	417
S7	(MM “Qualitative Studies+”) OR TI ((“focus group⁣^∗^” or qualitative or ethnograph⁣^∗^ or fieldwork or “field work” or “key informant” or interview⁣^∗^)) OR AB ((“focus group⁣^∗^” or qualitative or ethnograph⁣^∗^ or fieldwork or “field work” or “key informant” or interview⁣^∗^)) OR TI ((“semi-structured” or semistructured or unstructured or informal or “in-depth” or indepth or “face-to-face” or structured or guide) N3 (interview⁣^∗^ or discussion⁣^∗^ or questionnaire⁣^∗^)) OR AB ((“semi-structured” or semistructured or unstructured or informal or “in-depth” or indepth or “face-to-face” or structured or guide) N3 (interview⁣^∗^ or discussion⁣^∗^ or questionnaire⁣^∗^))	308,696
S6	S1 OR S2 OR S5	2757
S5	S4 AND S3	2018
S4	(MM “Diabetes Mellitus+”) OR TI Diabetes OR AB Diabetes	203,715
S3	(MM “Psychological Distress”) OR TI ((“psych⁣^∗^ distress” OR “emotional distress” OR “anxiety”)) OR AB ((“psych⁣^∗^ distress” OR “emotional distress” OR “anxiety”))	91,115
S2	TI ((“diabetes related” OR “diabetes specific”) N5 (distress⁣^∗^ OR stress⁣^∗^ OR concern⁣^∗^ OR problem⁣^∗^ OR conflict⁣^∗^ OR anxi⁣^∗^)) OR AB ((“diabetes related” OR “diabetes specific”) N5 (distress⁣^∗^ OR stress⁣^∗^ OR concern⁣^∗^ OR problem⁣^∗^ OR conflict⁣^∗^ OR anxi⁣^∗^))	583
S1	TI ((“diabetes distress” OR (diabetes N5 distress)) OR AB ((“diabetes distress” OR (diabetes N5 distress))	780

**Table 3:** McMaster Critical Review—quality appraisal of included studies.

**Study**	**54**	**46**	**41**	**47**	**50**	**44**	**42**	**51**	**37**	**38**	**53**	**40**	**48**	**39**	**49**	**13**	**45**	**52**	**43**
Study purpose																			
The purpose and/or research question was stated clearly	✓	✓	✓	✓	✓	✓	✓	✓	✓	✓	✓	✓	✓	✓	✓	✓	✓	✓	✓
Literature																			
Relevant background and literature were reviewed	✓	✓	✓	✓	✓	✓	✓	✓	✓	✓	✓	✓	✓	✓	✓	✓	✓	✓	✓
Study design																			
A theoretical perspective was identified	NR	NR	NR	✓	NR	✓	NR	✓	NR	NR	NR	NR	NR	NR	NR	NR	NR	NR	NR
Sampling																			
The process of purposeful selection was described	✓	✓	✓	✓	✓	✓	✓	✓	✓	✓	✓	✓	✓	✓	✓	✓	✓	✓	✓
Sampling was done until redundancy in data was reached	NR	NR	✓	✓	NR	NR	NR	NR	NR	NR	NR	NR	NR	NR	NR	✓	NR	NR	NR
Informed consent obtained	✓	✓	✓	✓	✓	✓	NR	✓	NR	NR	✓	✓	✓	✓	NR	NR	✓	NR	✓
Data collection																			
Clear and complete description of site	X	✓	✓	✓	✓	✓	✓	✓	✓	✓	✓	X	✓	✓	X	X	✓	✓	X
Clear and complete description of participants	✓	✓	✓	✓	✓	✓	✓	✓	✓	✓	✓	✓	✓	✓	✓	✓	✓	✓	✓
Role of researcher and relationship with participants	X	✓	✓	X	X	✓	✓	X	✓	✓	✓	NR	✓	X	NR	✓	✓	NR	X
Identification of assumptions and biases of researcher	NR	✓	NR	NR	NR	NR	NR	NR	NR	NR	NR	NR	✓	NR	NR	✓	✓	NR	NR
Procedural rigor was used in data collection strategies	X	✓	✓	✓	X	✓	✓	✓	✓	✓	✓	X	✓	X	✓	✓	✓	X	X
Data analysis																			
Data analyses were inductive	✓	✓	✓	✓	✓	✓	✓	✓	✓	✓	✓	✓	✓	✓	✓	✓	✓	✓	✓
Findings were consistent and reflective of data	✓	✓	✓	✓	✓	✓	✓	✓	✓	✓	✓	✓	✓	✓	✓	✓	✓	✓	✓
Audit trail was developed	X	✓	✓	✓	✓	X	✓	X	X	X	X	✓	✓	X	✓	✓	✓	✓	X
The process of analysing the data was described adequately	X	✓	✓	✓	✓	✓	X	✓	✓	✓	✓	✓	✓	✓	✓	✓	✓	✓	✓
A meaningful picture of the phenomenon under study emerged	X	✓	✓	✓	✓	✓	✓	✓	✓	✓	✓	✓	✓	✓	✓	✓	✓	✓	✓
Overall rigor																			
The research demonstrated the four components of trustworthiness:																			
Credibility	✓	✓	✓	✓	X	✓	✓	✓	✓	✓	✓	✓	✓	✓	✓	✓	✓	✓	✓
Transferability	X	✓	✓	✓	✓	✓	✓	✓	✓	✓	✓	X	✓	X	✓	X	✓	✓	✓
Dependability	X	✓	✓	✓	✓	X	X	X	X	X	X	✓	✓	X	✓	✓	X	X	X
Confirmability	X	✓	✓	✓	✓	X	X	X	X	X	X	✓	✓	X	✓	✓	X	X	X
Study conclusion and implications																			
Conclusions were appropriate given the study findings	✓	✓	✓	✓	✓	✓	✓	✓	✓	✓	✓	✓	✓	✓	✓	✓	✓	✓	✓
Findings contributed to theory development and future practice/research	✓	✓	✓	✓	✓	✓	✓	✓	✓	✓	✓	✓	✓	✓	✓	✓	✓	✓	✓

*Note:* X = some reporting of the criteria but did not match all the components, ✓ = criteria has been reported and done so adequately.

Abbreviation: NR = no reporting of the criteria.

**Table 4:** Distribution of descriptive themes across papers.

	**Citation number**																		
	**54**	**46**	**41**	**47**	**50**	**44**	**42**	**51**	**37**	**38**	**53**	**40**	**48**	**39**	**49**	**13**	**45**	**52**	**43**
Descriptive themes																			
Diabetic identity		x	x	x					x	x	x	x	x	x				x	
Stigma	x	x	x		x			x				x	x	x	x	x			
Fear of complications and the future	x	x	x		x	x	x		x	x	x		x	x	x	x	x	x	x
Too little support		x	x	x	x	x	x		x	x	x	x	x	x	x	x	x	x	x
Dominative support	x	x	x	x		x	x		x	x	x		x		x	x			
Diabetes as a barrier	x	x	x	x	x	x	x	x	x	x	x		x		x	x	x	x	x
Management issues and difficulties	x	x	x	x	x	x	x	x	x	x	x	x	x	x	x	x	x	x	x

**Table 5:** Data extraction of included studies.

**Paper** **Author, year (country)**	**Aim**	**Sample demographics**	**Data collection method**	**Data analysis approach**	**Key findings**
“You cannot cure it, just control it”: Jamaican adolescents living with diabetes Anderson and Tulloch-Reid, 2019 [54] (Jamaica)	To investigate the experiences of Jamaican adolescents living with diabetes to determine how their needs can be addressed	Nineteen participants—15 with T1DM and four with T2DM (average age 14)	Focus groups (discussion and drawing)	Thematic analysis to analyse narratives. Drawings analyzed using a variation of Lauritsen and Mathiasen's (2003)	• Children feel controlled by their condition, they feel restricted in activities which leads to a sense of difference. • Support from others can either be interpreted as helpful or overbearing. • Children would try to break the rules and be secretive in attempts to resist control. • Children living in rural areas experienced more distress than those in urban areas.

‘Diabetes is a gift from god' a qualitative study coping with diabetes distress by Indonesian outpatients Arifin et al., 2020 [46] (Indonesia)	Explore distress and coping strategies in Indonesian T2DM outpatients in a primary healthcare centre (PHC) in Surabaya, East Java, Indonesia	Forty-three participants with T2DM (≥ 18)	Focus group discussions and semistructured interviews	Thematic analysis	• Internal sources of DD consist of disease burden, fatigue due to T2DM, fatigue not due to T2DM, emotional burden (fear, anxiety, etc.), and lack of knowledge. Internal coping strategies comprised spirituality, a positive attitude, acceptance, and getting more information about T2DM. • DD that comes from external sources typically centres around healthcare services, diet, routine medication, monthly blood sugar checks, interpersonal distress (family), and financial concerns.

What's distressing about having type 1 diabetes? A qualitative study of young adults' perspectives Balfe et al., 2013 [41] (Ireland)	To identify causes of DD in a sample of young adults with T1DM	Thirty young adults with T1DM (aged 23–30 years)	Semistructured interviews	Thematic analysis	• Multiple factors can trigger DD, most commonly self-consciousness, day-to-day management difficulties, tensions with the healthcare system, concerns about the future, and apprehension about pregnancy. • Techniques to help moderate DD in this sample included opportunities to talk to healthcare professionals about DD and education programmes.

Exploring the perceptions of emotional distress among couples living with type 2 diabetes and among diabetes healthcare providers, and consideration of support needs Berry, Davies, and Dempster, 2020 [47] (United Kingdom)	Explore distress and coping strategies in Indonesian T2DM outpatients in a primary healthcare centre (PHC) in Surabaya, East Java, Indonesia	Twenty-two adults (age ≥ 18 years) with T2DM	Open interviews	Inductive thematic analysis	• Unique psychological struggles for people with Type 2 diabetes included “sense of restriction,” “disempowerment,” and “acceptance of diabetes.” • Common themes across people with T2DM, partners, and healthcare providers included “self-care struggles” and “perceived need for appropriate psychological support.”

General life and diabetes-related stressors in early adolescents with type 1 diabetes Chao et al., 2016 [50] (United States)	To examine general life and diabetes-specific stressors from the perspective of early adolescents (ages 11–14) with T1DM	Two hundred five adolescences with T1DM	Adolescents were asked to identify their Top 3 stressors from a checklist and respond to open-ended questions about what was stressful about each of their Top 3 stressors	Content analysis	• Eight themes were found overall. Five of these are related to general life stressors (fitting in, having friends, balancing competing demands, living with family, and feeling pressure to do well). The remaining themes are related to diabetes-specific stressors (just having diabetes, dealing with emotions, and managing diabetes).

The lived experience of older adults with type 2 diabetes mellitus and diabetes-related distress Hernandez, 2017 [44] **(**United States)	The purpose of this phenomenological study was to understand and describe how diabetes distress might affect older adults (age 65 years and older) with T2DM	Sixteen participants (between the ages of 65 and 85) with T2DM	Interpretive interviews	Narrative and thematic analyses	• Unsatisfactory relationships with healthcare providers were a prominent issue. • Participants felt guilty about lifestyle choices which may have led to the development of diabetes. • Forgetfulness and fatigue negatively impacted self-care routines. • Hypoglycaemia was noted to lead to falls and loss of consciousness. • Diarrhoea, often induced by medications, interfered with day-to-day life. • Pain resulted in sleep disruption and worsened fatigue.

Understanding diabetes-related distress characteristics and psychosocial support preferences of urban African American adults living with type 2 diabetes a mixed-methods study Hood et al., 2018 [42] (United States)	To explore what aspects of T2DM cause the most distress among African Americans living with the condition	Twenty-three Urban African Americans (aged ≥ 18 years)	Focus groups	Thematic analysis	• Primary aspects of DD were related to regiment distress and emotional burden. • Participants prefer culturally appropriate peer support.

Culturally meaningful leisure as a way of coping with stress among aboriginal individuals with diabetes Iwasaki and Bartlett, 2006 [51] (Canada)	Gain insight into the lived experiences of urban aboriginal Canadians with diabetes in stress and coping through leisure	Twenty-six aboriginal individuals (aged 26–69 years) with unspecified diabetes	Focus groups	Phenomenology	• Participants experiences of stress were linked to the diabetes-related aspects of their lives. • Description of stress was also rooted in broader structural systems (socioeconomic, cultural, and historic).

Social support and self-management of type 2 diabetes among immigrant Australian women Kokanovic and Manderson, 2005 [37] (Australia)	To understand the social meanings and interpretations that immigrant women attach to the diagnosis of T2DM and the social support and professional advice that they receive following this diagnosis	Sixteen immigrant women living in Melbourne, Australia, with T2DM (≥ 18 years)	Interviews	Thematic analyses	• Some participants were critical of their clinicians but also manipulated their encounters to avoid receiving unwelcome information about the progress of the disease changes in the regiment. • Social interactions influenced women's adjustments to diagnosis. • Participants highlighted a fine line between support and interference. • Talking to others with the condition was beneficial as participants felt that only others with the condition understood what it was like.

“Worried all the time”: distress and the circumstances of everyday life among immigrant Australians with type 2 diabetes Manderson and Kokanovic, 2008 [38] (Australia)	To explore how lay accounts of distress and depression relate to both the chronicity of diabetes and the circumstances of everyday life and the coincidence of distress, depression, and physical illness	Thirty-nine immigrant individuals living with T2DM in Australia (≥ 18 years)	Open-ended interviews	Thematic analysis	• Participants generally viewed stress as a precursor to diabetes. They also believed this influenced their ability to control symptoms. • Participants regarded diabetes as an illness that interrupted their ability to carry out day-to-day tasks.

The psychological impact of living with diabetes women's day-to-day experiences Penckofer et al., 2007 [53] (United States)	To understand the feelings of depression, anxiety, and anger experienced by women with Type 2 diabetes and the impact these feelings have on their overall quality of life.	Forty-one women with T2DM (average age 55.6 years)	Focus groups	Content analysis	• Five themes were extracted struggling with the changing health situation; encountering challenges in relationships with self, family, and others; worrying about the present and future; bearing multiple responsibilities for self and others; and choosing to take a break. • Women expressed emotions such as depression, anxiety, and anger linked to having DM and managing the multiple responsibilities of being a caregiver.

“Anxiety and type 1 diabetes are like cousins”: the experience of anxiety symptoms in youth with type 1 diabetes Rechenberg, Grey, and Sadler, 2018 [40] (United States)	Describe the experience of anxiety symptoms and anxiety-related sleep disturbance in youth with T1DM, especially as those symptoms related to daily tasks associated with the diabetes treatment regiment	Participants (*n* = 29, ages 10–16)	Semistructured interviews	Thematic analyses	• T1DM was viewed as an added layer of responsibility that limited aspects of their lives. • Two types of anxiety were identified, general and diabetes-specific described. • Some participants were able to integrate diabetes management into their lives, while others were not.

Contextualizing experiences of diabetes-related stress in rural Dominican Republic Gonzalez Rodriguez, Wallace, and Barrington, 2019 [48] (Dominican Republic)	To explore the emotional burden of T2D and identify coping strategies	Twenty-eight participants (aged ≥ 18 year) with T2DM	Interviews	Inductive analysis	• Stress relating to T2DM began at diagnosis and persisted throughout management. • Stress was caused by a range of concerns, including, healthy food and medication access, fears about illness-induced injury, and the cyclical process of experiencing stress. • Stress could be reduced externally through diabetes care and free medication services or internally mitigated by not thinking about diabetes (“no dar mente”)

Socio-psychological problems of patients with late adolescent onset type 1 diabetes--analysis by qualitative research Sato et al., 2003 [39] (Japan)	To identify the serious emotional impacts as sociopsychological problems encountered by patients with late adolescent-onset Type 1 diabetes since their disease onset	Thirteen participants (aged 21–35 years) with T1DM	Semistructured interviews	Thematic analysis	• Problems that were identified related to initial diagnosis, negative emotions associated with insulin injections, unexpected hypoglycaemic events, not complying with the diet, and stress of disclosing the disease.

Personal accounts of the negative and adaptive psychosocial experiences of people with diabetes in the second diabetes attitudes, wishes and needs (DAWN2) study Stuckey et al., 2014 [49] (17 countries included)	To identify the psychosocial experiences of diabetes, including negative accounts of diabetes and adaptive ways of coping from the perspective of the person with diabetes	Eight thousand five hundred ninety-six T1DM and T2DM (aged ≥ 18years)	Open-ended survey questions	Thematic content analyses	• People with diabetes experience anxiety/fear, concerns about hypoglycaemia and complications of the condition, negative moods, hopelessness, public misunderstanding of diabetes, and discrimination at work.

Diabetes distress from the patient's perspective: qualitative themes and treatment regimen differences among adults with type 2 diabetes Tanenbaum et al., 2016 [13]	To explore diabetes distress in a sample of adults with Type 2 diabetes, treated and not treated with insulin.	Thirty-two participants (aged ≥ 18 years) with T2DM	Focus groups	Thematic analysis	• A range of sources of DD were identified, including lack of support/understanding from others, difficulties communicating with providers, and distress from the burden of lifestyle changes. Participants using insulin described significant emotional distress related to the burden of their insulin regimen and were more likely to report physical burden related to diabetes; to describe feeling depressed as a result of diabetes; and to express distress related to challenges with glycaemic control. • Alternatively, noninsulin-treated participants were more likely to discuss the burden of comorbid medical illnesses.

Diabetes: a cross-cultural interview study of immigrants from Somalia Wallin, Löfvander, and Ahlström, 2007 [45] (Sweden)	To describe how diabetic immigrants from Somalia experience everyday life in Sweden and how they manage diabetes-related problems, with the inclusion of a gender perspective	Nineteen Somalian participants with T2DM (aged ≥ 18 years)	Interviews	Content analysis	• Four themes emerged: experience of distress in everyday life, everyday life continues as before, comprehensibility giving a feeling of control, and being compliant. • Participants varied in how they managed fasting during Ramadan, with some not seeing diabetes as an obstacle while others highlighted that it is not necessary for a sick person to fast.

The distress experienced by people with type 2 diabetes West and Mcdowell, 2003 [52] (United Kingdom)	Understand what the diabetes-related distress of Type 2 diabetes in men and women	Ten participants with T2DM (aged ≥ 18 years)	Focus groups	Morse's (1991) qualitative analysis	• Three broad categories were identified related to dietary management: dietary restrictions, value judgments, and the influence of others. • Behavioural impact, emotional impact, and fear of complications were major themes identified in the focus groups.

When qualitative data contradict quantitative data: diabetes distress in the Chinese-Canadian community Xia, Yau, and Tang, 2018 [43] (Canada)	To use both quantitative and qualitative approaches to characterize the diabetes distress profile of Chinese-Canadians with T2DM and to better understand their experience of living with diabetes	Forty Chinese-Canadian adults with T2DM (aged ≥ 18 years)	Semistructured interviews	Immersion–crystallization approach	• Participants were dissatisfied with their diabetes care providers and experienced emotional challenges, eating distress, fear of complications, language barriers, and medication concerns.

Abbreviations: NR = not reported, T1DM = Type 1 diabetes mellitus, T2DM, Type 2 diabetes mellitus.

## Data Availability

The qualitative data supporting this systematic review are from previously reported studies and datasets, which have been cited. The processed data are available from the corresponding author upon request.

## Supporting Information

Supporting Information Additional supporting information can be found online in the Supporting Information section. Supporting Information 1 contains a table showcasing an example of how descriptive themes were developed by grouping initial codes.
