# Supplementary material for: Understanding Experiences of Diabetes Distress: A Systematic Review and Thematic Synthesis
Source: J Diabetes Res. 2024 Nov 14;2024:3946553. doi: 10.1155/2024/3946553 (PMC11581805; doi:10.1155/2024/3946553)
Supplement: Supporting Information — Additional supporting information can be found online in the Supporting Information section. Supporting Information 1 contains a table showcasing an example of how descriptive themes were developed by grouping initial codes. [file 3946553.f1.docx]

**Theme: Stigma**

| *Overarching Code* | *2^nd^ stage code (if applicable)* | *1^st^ stage code* | *Example Quotation* |
| --- | --- | --- | --- |
| **Stigma and discrimination** | Work-placed discrimination | Fired due to diabetes | When one man told his employer about having diabetes, after previously having hidden taking his medications, he was ﬁred |
|  |  | Employers not hiring diabetics | “When I went to get a job, I was not hired as soon as they found out about my illness. They said that hiring an employee with the disease is not economically justiﬁable, as it leads to frequent medical leaves.”50 |
|  |  | Work discrimination | “I was written up at work for arriving late. I was unable to drive due to low blood sugar and demoted into another position so my health problems wouldn't 'get in the way” |
|  |  | Employers not hiring diabetics | “When I went to get a job, I was not hired as soon as they found out about my illness. They said that hiring an employee with the disease is not economically justiﬁable, as it leads to frequent medical leaves.” |
|  | Public ill-informed about diabetes | Lack of Public understanding | “People are like, ‘oh which kind do you have? Do you have the really bad kind?’ I’m like, ‘what do you mean by that?’ |
|  |  | Need for increased public awareness | These experiences of discrimination are set against the “wish for improvement for increased public awareness” |
|  |  | Attempting to educate people | “I try to use these moments to teach them about how their comments are misinformed and downright rude, yet I feel it falls on deaf ears” |
|  |  | People not appreciating the extent of illness | “I don’t think people really understand what’s involved” |
|  |  | Benefits of improved public knowledge | Others mentioned that increased knowledge about diabetes may reduce prejudice against people with diabetes. |
|  |  | Negative media representations | “People are apt to make a fun of diabetes. For example, on TV, the disease is often used for laughs, such as “If you eat sweets so much, you will have diabetes!” I wonder why people have such a poor image of the disease” |
|  |  | Better media representation | “I want to have the media widely publicize insulin injections in the society,” |
|  | Stigma by association | Us vs them (T1 V T2) | “That’s something that drives me crazy, Type 1 and 2 diabetes. It makes me so annoyed. Type 1 diabetes, you don’t get it because you’re overweight.” |
|  |  | T1DM Dislike for T2DM | These interviewees themselves appeared to have negative perspectives of Type 2 diabetes; they seemed to see themselves as risk-avoidant, responsible subjects who developed diabetes through no fault of their own, whereas they thought that people with Type 2 developed their diabetes as a result of moral failings (i.e. inabilities to control their bodies and appetites) |
|  |  | Misidentification of having T2DM | Just under half of interviewees (n = 15) described feeling angry and frustrated at Type 2 diabetes. There were two reasons for this anger. The first was that interviewees felt that there a strong risk that they themselves could be misidentified as having Type 2 diabetes. |
|  |  | Unsolicited comments | “People feel like they can say anything to you … like they can make comments like ‘You ought to watch what you eat.’ I mean total strangers, plus your well-meaning friends who say, ‘Do you think you should be eating that?” |
|  |  | Stigma | Some feared what they perceived as the “stigma” associated with diabetes—“I find hard to express to people that I have diabetes, and I try to keep it hidden so that most people don’t find out, because I feel like they will judge me.” |
|  |  | Discrimination | “People make fun of me, not being able to do everything I would wish” |
|  |  | Ashamed of diabetes | “This is not something to brag about/I hide it” |
|  |  | Diabetes imposing difference | They described having diabetes as something that made them different |
|  |  | Diabetes viewed as defect by others | One woman explained that she did not take insulin in front of her boyfriend’s parents because diabetes was viewed as a defect, which was unacceptable |
|  |  | Being diabetic is seen as bad | “There are people who interpret [diabetes] as bad” |
|  |  | Disclosure | “I, instead, try to avoid telling people because I do not want other people to consider me invalidated by this disease” |
| **Distress of visibility and management of diabetes in public** | Wanting to hide diabetes | Visibility of diabetes management | Diabetes management had a visible component that other people could easily see, related to diet and injections. |
|  |  | Hide management | Some refused to wear insulin pumps or continuous glucose monitors because they could be seen and heard. |
|  |  | Diabetes equipment drawing attention | “I don’t need, like, something beeping during the day, like, drawing all attention to me” |
|  |  | Diabetes being visible | “People can see it, I think that’s the worst thing” |
|  |  | Not wanting diabetes to be noticed | “There are occasions when it takes time to do my regular insulin injection without being noticed by others. I wish there was a medication for diabetes that could be taken without being noticed.” |
|  |  | Feeling uncomfortable managing diabetes in public | Some felt ‘awkward’ when they had to manage their diabetes around others. |
|  |  | Publicly injecting | Some people with diabetes felt uncomfortable giving injections in public and were ashamed to be caught injecting as if a “drug user.” |
|  |  | Self-Consciousness | “I feel a bit uncomfortable. I don’t know the people that well inside there.” |
|  |  | Feeling uncomfortable managing diabetes in front of new people | These were generally situations where interviewees either lost access to previously supportive audiences who could help to (re)frame diabetes management as a ‘normal’ activity, or forced interviewees to manage their diabetes in front of new and (potentially) unsupportive audiences. |
